# Conjugation of Human β-Defensin 2 to Spike Protein Receptor-Binding Domain Induces Antigen-Specific Protective Immunity against Middle East Respiratory Syndrome Coronavirus Infection in Human Dipeptidyl Peptidase 4 Transgenic Mice

**DOI:** 10.3390/vaccines8040635

**Published:** 2020-11-01

**Authors:** Ju Kim, Ye Lin Yang, Yongsu Jeong, Yong-Suk Jang

**Affiliations:** 1 Department of Molecular Biology and the Institute for Molecular Biology and Genetics, Jeonbuk National University, Jeonju 54896, Korea; ju226@jbnu.ac.kr; 2 Department of Bioactive Material Sciences and Research Center of Bioactive Materials, Jeonbuk National University, Jeonju 54896, Korea; yyr7637@jbnu.ac.kr; 3 Graduate School of Biotechnology, Kyung Hee University, Yongin 17104, Korea; yongsu@khu.ac.kr

**Keywords:** adjuvant, antibody, defensin, MERS-CoV, vaccine

## Abstract

Middle East respiratory syndrome coronavirus (MERS-CoV) causes severe acute respiratory symptoms. Due to the lack of medical countermeasures, effective and safe vaccines against MERS-CoV infection are urgently required. Although different types of candidate vaccines have been developed, their immunogenicity is limited, and the dose and administration route need optimization to achieve optimal protection. We here investigated the potential use of human β-defensin 2 (HBD 2) as an adjuvant to enhance the protection provided by MERS-CoV vaccination. We found that immunization of human dipeptidyl peptidase 4 (hDPP4)-transgenic (hDPP4-Tg) mice with spike protein receptor-binding domain (S RBD) conjugated with HBD 2 (S RBD-HBD 2) induced potent antigen (Ag)-specific adaptive immune responses and protected against MERS-CoV infection. In addition, immunization with S RBD-HBD 2 alleviated progressive pulmonary fibrosis in the lungs of MERS-CoV-infected hDPP4-Tg mice and suppressed endoplasmic reticulum stress signaling activation upon viral infection. Compared to intramuscular administration, intranasal administration of S RBD-HBD 2 induced more potent mucosal IgA responses and was more effective for protecting against intranasal MERS-CoV infection. In conclusion, our findings suggest that HBD 2 potentiates Ag-specific immune responses against viral Ag and can be used as an adjuvant enhancing the immunogenicity of subunit vaccine candidates against MERS-CoV.

## 1. Introduction

Since 2000, numerous potentially lethal zoonotic human diseases have emerged due to novel coronaviruses, including severe acute respiratory syndrome coronavirus (SARS-CoV) in 2002, Middle East respiratory syndrome coronavirus (MERS-CoV) in 2012, and SARS coronavirus 2 (SARS-CoV-2) in 2019. Owing to their pandemic potential, impact on global health, and lack of effective medical countermeasures, these coronaviruses have been included in the World Health Organization (WHO) Research and Development Blueprint list of priority diseases. The MERS-CoV outbreak in Korea in 2015 has caused significant morbidity and mortality, posing severe threats to public health and the economy [1]. Particularly, Saudi Arabia still has the highest reported MERS-CoV mortality rate with approximately 80% [2]. Despite extensive research efforts to develop effective prophylactic or therapeutic interventions against MERS-CoV, no treatments or vaccines have gained regulatory approval thus far.

Most MERS-CoV vaccines under investigation involve inactivated virus, live attenuated virus, viral vector-based vaccines, recombinant virus subunits, and DNA vaccines [3]. The most common MERS-CoV vaccine targets are the spike (S) protein or the S protein domain required for binding to the host receptor, dipeptidyl peptidase 4 (DPP4); however, a variable degree of immunogenicity has been reported with these vaccines. Thus, the use of proper adjuvants in addition to the optimization of the dose and administration route is imperative to induce potent and long-lasting protective immunity [4,5,6]. In particular, co-administration of adjuvants with MERS-CoV S protein in mice induced the production of neutralizing antibodies (Abs) [7]. Alum is a commonly used adjuvant in vaccine formulations, as it enhances the production of antigen (Ag)-specific Abs and induction of cellular immunity [8]. However, alum alone is inefficient to induce potent type 1 helper T (Th1) responses and subsequent viral clearance unless combined with additional adjuvants, such as glucopyranosyl lipid [9]. Therefore, the combination of potent Ag and adjuvants capable of mimicking a natural infection inducing strong primary immune responses is effective for the induction of antiviral immune responses against MERS-CoV.

Host defense peptides play important roles as primary gatekeepers protecting respiratory, oral, reproductive, and enteric tissues from various pathogens and maintaining tissue homeostasis [10,11]. Among them, human β-defensins (HBDs) are small host defense peptides expressed by epithelial cells and establish mucosal barriers against various infectious agents. In addition to their antimicrobial roles, HBDs link the activation of pathogen-specific innate and adaptive immunity through the recruitment and activation of various leukocytes, including macrophages, dendritic cells (DCs), and T cells [12,13,14]. We previously reported that HBD 2 promotes antiviral innate immune responses in macrophage-like THP-1 cells and enhances Ag-specific responses in vivo after immunization with the receptor-binding domain (RBD) of MERS-CoV S protein (S RBD) [15]. More importantly, conjugation of Ag to HBD 2 enhances type I immune responses by promoting macrophage activation and polarization, ultimately leading to more potent Ag-specific adaptive immune responses [16].

MERS-CoV infects the lower respiratory tract, leading to severe acute respiratory failure and progressive pulmonary fibrosis. Small experimental animals, such as mice, ferrets, and hamsters, are non-permissive for MERS-CoV infection due to structural differences in DPP4 at the interface with MERS-CoV S RBD. We previously generated a human DPP4-transgenic (hDPP4-Tg) mouse model for MERS-CoV infection [17]. Pulmonary fibrosis is characterized by fibroblast proliferation and extracellular matrix remodeling leading to respiratory insufficiency. Nevertheless, the mechanisms involved in lung fibrosis following MERS-CoV infection remain poorly understood. Herein, using the hDPP4-Tg mouse model, we show that MERS-CoV infection activates endoplasmic reticulum (ER) stress pathway components, including protein kinase RNA-like ER kinase (PERK), activating transcription factor 4 (ATF4), C/EBP homologous protein (CHOP), and activating transcription factor 6 (ATF6). We also show that the administration of HBD 2-conjugated S RBD (S RBD-HBD 2) reduced ER stress signaling and alleviated progressive pulmonary fibrosis in the lungs of MERS-CoV-infected hDPP4-Tg mice. In addition, we provide evidence that the intranasal administration of S RBD-HBD 2 is more effective in inducing mucosal IgA responses and protective immunity against MERS-CoV.

## 2. Materials and Methods

### 2.1. Materials and Experimental Animals

hDPP4-Tg mice were generated as previously reported [17], and all mice were bred and maintained at Kyung Hee University (Suwon, Korea). Male and female hDPP4-Tg mice (5–7 weeks old) were used for experiments; they were housed under specific pathogen-free conditions with water and food provided *ad libitum*. All animal experiments were approved by the Institutional Animal Care and Use Committee of Jeonbuk National University (Approval No. CBNU 2018–049) and performed in accordance with the committee’s guidelines. MERS-CoV (1–001-MER-IS-2015001) was obtained from the Korean Center for Disease Control and Prevention (KCDC). All experiments using MERS-CoV were performed in accordance with the WHO’s recommendations under biosafety level 3 conditions in a biosafety level 3 facility of the Korea Zoonosis Research Institute (Iksan, Korea), Jeonbuk National University. Unless otherwise specified, the chemicals and laboratory wares used in this study were obtained from Sigma Chemical Co. (St. Louis, MO, USA) and SPL Life Sciences (Pocheon, Korea), respectively.

### 2.2. Recombinant Protein Production, Mouse Immunization, and Sample Collection

Production of the recombinant MERS-CoV S RBD with or without HBD 2 at the C terminus of the S1 (residues 291–725) domain based on the MERS-CoV S protein sequence (GenBank: AKL59401.1; GenScript, Piscataway, NJ, USA) was performed as described previously [15,18].

hDPP4-Tg mice were immunized intramuscularly in the hind leg with 5 µg/mouse of each recombinant protein dissolved in 50 µL phosphate-buffered saline (PBS) emulsified with an equal volume of Freund’s complete adjuvant. Ten days after the first immunization, mice were boosted with the same immunogen emulsified with Freund’s incomplete adjuvant. In addition, hDPP4-Tg mice were intranasally immunized once per week for five weeks with 1 µg each recombinant protein via the intranasal route under anesthesia. Control mice were immunized with the inoculum prepared identically but with PBS only. Sera were collected three days after the final immunization boost to measure MERS-CoV S RBD-specific Abs.

### 2.3. Enzyme-Linked Immunosorbent Assay (ELISA)

The levels of MERS-CoV S RBD-specific immunoglobulin G (IgG) in mouse sera were determined by ELISA. Briefly, 96-well ELISA plates (Thermo Fisher Scientific, Waltham, MA, USA) were coated with S RBD protein (2 µg/mL) overnight at 4 °C and blocked with 5% nonfat dry milk at 37 °C for 2 h. For preparing the standard curve, the anti-mouse IgG coating antibody was coated onto an ELISA plate, unlike the serum sample. After adding serially diluted sera or standard mouse IgG to each well, plates were incubated at 37 °C for 1 h, followed by four washes with phosphate-buffered saline (PBS) containing Tween 20. Bound IgGs were incubated with alkaline phosphate-conjugated anti-mouse IgG at 37 °C for 1 h, and *p*-nitrophenyl phosphate substrate was added. The absorbance at 405 nm was read using an ELISA plate reader (SPECTROstar Nano, BMG Labtech, Ortenberg, Germany). ELISA results were calculated using the standard curve.

### 2.4. Viral Challenge and Sample Collection

MERS-CoV was propagated in Vero E6 cells grown in Dulbecco’s Modified Eagle’s Medium (Welgene, Gyungsan, Korea) supplemented with 10% fetal bovine serum (Thermo Fisher Scientific) at 37 °C in a humidified CO_2_ incubator. MERS-CoV was passaged 12 times in Vero E6 cells and subsequently used to assess hDPP4-Tg mouse morbidity and mortality. Briefly, hDPP4-Tg mice and their transgene-negative littermates were anesthetized and inoculated intranasally with 10^4^ or 10^5^ plaque-forming unit (PFU) of MERS-CoV in a total volume of 20 µL. Infected mice were weighed and monitored every other day for weight loss and death. Although the scoring was not recorded, other clinical signs following viral infection, including physical appearance, abnormalities of behavior or movements, and decreased activity or enhanced responsiveness, were also observed. Subsequently, immunized hDPP4-Tg mice were challenged with 10^5^ PFU of MERS-CoV intranasally and monitored for their survival, weight, and pathological changes for up to 10 or 14 days post-infection (dpi). Some infected mice were sacrificed at the indicated time points to obtain tissue specimens. These tissue specimens were used to assess the expression levels of target genes and mucosal IgA responses using real-time quantitative reverse transcription-polymerase chain reaction (qRT-PCR) and hematoxylin-eosin (H&E) staining.

### 2.5. Histopathology

Lung tissues obtained from MERS-CoV- and sham-infected hDPP4-Tg mice at the indicated time points were immediately fixed in 10% neutral buffered formalin, transferred to 70% ethanol, and paraffin-embedded. Histopathological evaluation was performed on tissue sections deparaffinized and stained by H&E. We examined tissues for pathological signs, such as denatured and collapsed cell/tissue organization, hemorrhage in the interstitial space, infiltration of inflammatory monocytes, and change in alveolar septa after MERS-CoV infection.

### 2.6. RNA Extraction and qRT-PCR

Lung tissue specimens from MERS-CoV-infected mice were weighed and transferred into individual vials containing TRIzol reagent (Thermo Fisher Scientific). The collected tissues were homogenized and subjected to total RNA isolation as previously described [16]; RNA was extracted using TRIzol reagent according to the manufacturer’s instructions. RNA was converted into cDNA using an MMLV Reverse Transcription Kit (Promega, Fitchburg, WI, USA). Gene expression analyses were performed using qRT-PCR with the SsoAdvanced Universal SYBR Green Supermix (Bio-Rad Laboratories, Hercules, CA, USA) and a CFX Connect Real-Time System (Bio-Rad Laboratories). For the reactions, 50 ng first-strand cDNA was used. The following qRT-PCR conditions were used: 95 °C for 5 min followed by 40 cycles at 95 °C for 15 s, 55 °C for 30 s, and 72 °C for 30 s. The relative expression level of each gene was obtained by normalizing it to that of the endogenous control gene β-actin; fold changes were calculated using the CFX Maestro software (Bio-Rad Laboratories). The gene expression level of the control group was set as a reference, whose value should be 1. The primers used for qRT-PCR are listed in Table 1.

### 2.7. Statistical Analyses

Statistical analyses were performed using Prism 7 (GraphPad, San Diego, CA, USA). Data are expressed as means ± standard deviations (SDs). The statistical significance of numerical data was analyzed using two-way analyses of variance (ANOVA). *p*-Values < 0.05 were considered statistically significant.

## 3. Results

### 3.1. MERS-CoV Infection in hDPP4-Tg Mice Causes Mortality and Morbidity with Progressive Pulmonary Fibrosis

**Figure 1:**
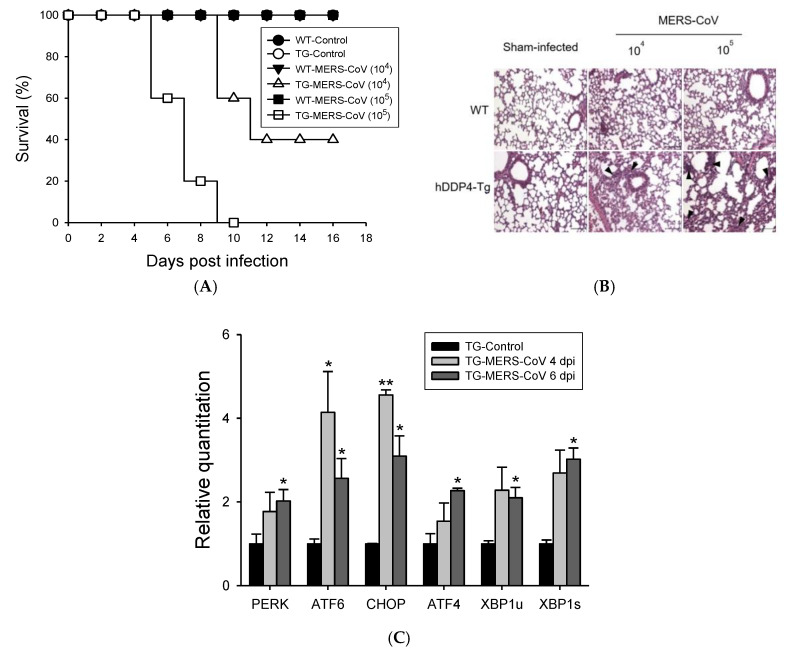
MERS-CoV infection in hDPP4-Tg mice causes mortality and morbidity with progressive pulmonary fibrosis. hDPP4-Tg mice and transgene-negative littermates were challenged intranasally with MERS-CoV (10^4^ or 10^5^ plaque-forming unit (PFU)) or phosphate-buffered saline (PBS). Infected mice were monitored every other day for weight loss, clinical symptoms, and survival. (**A**) Survival of hDPP4-Tg mice (TG) and transgene-negative littermates (WT) after infection with MERS-CoV or sham (PBS, control) (*n* = 10). (**B**) Histopathological changes in the lungs of hDPP4-Tg mice and transgene-negative littermates (WT) challenged with MERS-CoV or PBS (sham-infected). Ten days after MERS-CoV or sham infection, lung tissues were collected, fixed, and paraffin-embedded for hematoxylin and eosin staining. Hematoxylin and eosin (H&E)-stained lung sections were analyzed for inflammation by light microscopy. Transgene-negative littermates (WT) and sham-infected hDPP4-Tg mice represented normal lung tissue with thin-lined alveolar septa and well-architected alveoli. Virus-challenged groups showed distorted lung morphologies, including collapsed alveolar spaces with wider and thicker alveolar septa and perivascular and peribronchial cuffing. Regions of inflammatory cell infiltration around vasculature, bronchiole, and proximal alveoli were noted by arrowheads. Scale bars = 100 µm. (**C**) Effects of MERS-CoV infection in the expression of ER-stress-associated genes in the lungs of hDPP4-Tg mice. Total RNA was extracted from the lungs of hDPP4-Tg mice four and six days after infection with MERS-CoV. Relative expression of ER-stress-associated genes was determined by qRT-PCR after normalizing to *β-actin* mRNA levels. Reactions were performed in duplicates. Fold changes relative to non-treated controls are shown as means ± SD (*n* = 2). * *p <* 0.05 and ** *p <* 0.01.

Wild-type mice, including BALB/c and C57BL/6 mice, are not permissive to MERS-CoV infection [19]. Therefore, we previously generated hDDP4-Tg mice as a MERS-CoV infection model. To determine MERS-CoV mortality and morbidity in hDDP4-Tg mice, we intranasally inoculated hDPP4-Tg mice and age-matched transgene-negative littermates with 10^4^ or 10^5^ PFU of MERS-CoV. The mice were monitored every other day for survival and clinical symptoms, including weight loss (Figure 1A). hDPP4-Tg mice inoculated with 10^4^ or 10^5^ PFU of MERS-CoV showed rapid weight loss (data not shown); the mortality rates were 60% and 100% at 10 dpi and 12 dpi, respectively. All transgene-negative littermate mice survived without clinical illnesses after the inoculation of the same dose of MERS-CoV. In contrast to sham-infected mice, progressive lung damage was observed in MERS-CoV-infected hDPP4-Tg mice in a viral dose-dependent manner (Figure 1B). Importantly, MERS-CoV-infected hDPP4-Tg mice exhibited progressive pulmonary fibrosis signs, including an irregular arrangement of pneumocytes, alveolar septal thickening, and mild inflammation with infiltration of inflammatory cells into the lung at 10 dpi. However, no histopathological changes were detected in the lungs of sham-infected hDPP4-Tg mice or transgene-negative littermate mice.

Upon viral infection, large amounts of viral proteins are produced and accumulated in the ER, inducing ER stress [20]. Pulmonary viral infections often induce an ER-stress-mediated hyperinflammatory response, leading to fibrosis [21,22]. Here, we investigated whether ER stress contributes to lung fibrosis following MERS-CoV infection by analyzing the levels of ER-stress-associated genes, such as *Perk*, *Atf4*, *Chop*, *Atf6*, and X-box binding protein 1 (*Xbp1*) in the lungs of MERS-CoV-infected hDPP4-Tg mice and sham-infected hDPP4-Tg mice (Figure 1C). *Perk*, *Atf4*, and *Xbp1* were significantly upregulated (*p* < 0.05) in the lungs of MERS-CoV-infected hDPP4-Tg mice. Furthermore, MERS-CoV infection in hDPP4-TG mice significantly upregulated *Atf6* (*p* < 0.05) and *Chop* (*p* < 0.01) in lung tissues four dpi; however, their mRNA levels were reduced at six dpi. These results suggest that MERS-CoV infection in hDPP4-Tg mice leads to lung damage by triggering ER stress and fibrosis.

### 3.2. HBD 2-Conjugated Ag Elicits Potent Ag-Specific Ab Response in Hdpp4-Tg Mice Preventing MERS-CoV Infection

**Figure 2:**
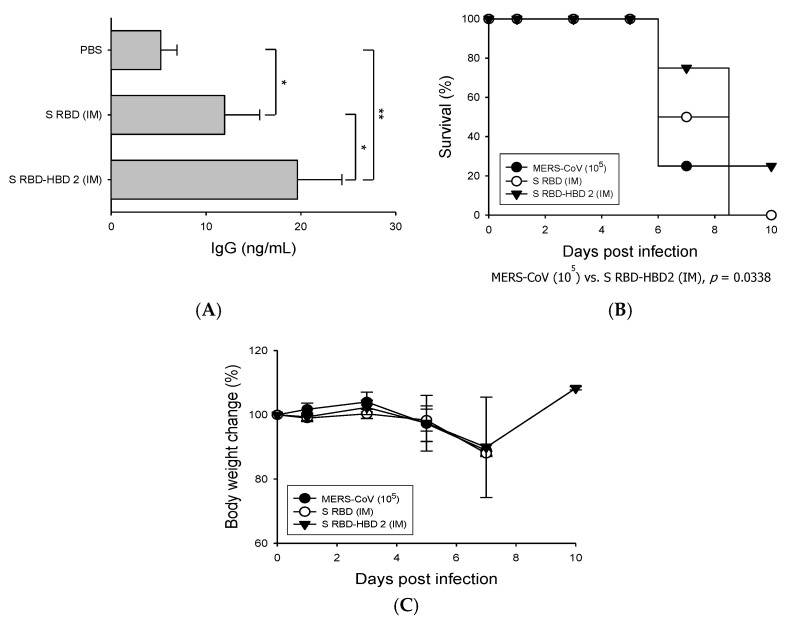
Immunization with S RBD-HBD 2 elicits potent Ag-specific immunity and reduces mortality upon MERS-CoV infection in hDPP4-Tg mice. Mice were intramuscularly (IM) immunized with 5 µg/mouse of S RBD with or without HBD 2, and sera were collected three days after boost immunization. The levels of S RBD-specific IgG were measured using enzyme-linked immunosorbent assay (ELISA). Data are presented as means ± SDs (*n* = 3). Immunized mice were challenged intranasally (IN) with MERS-CoV (10^5^ PFU). Infected mice were monitored every other day for weight loss, clinical symptoms, and survival. (**A**) Serum levels of S RBD-specific IgG which were determined via capture ELISA using standard mouse IgG. * *p* < 0.05 and ** *p* < 0.01. (**B**) Survival of hDPP4-Tg mice after MERS-CoV infection (*n* = 8). *P*-values were calculated using the log-rank (Mantel–Cox) test. (**C**) Bodyweight changes in MERS-CoV-infected mice. Results are presented as means ± SDs (*n* = 8) at the indicated times post-infection. There is no statistical significance in body weight changes among the groups tested.

Next, we investigated the immunogenicity of HBD 2-conjugated S RBD (S RBD-HBD 2) compared to that of S RBD alone by immunizing the mice intramuscularly (Figure 2). The levels of S RBD-specific IgG in the sera of mice immunized with S RBD-HBD 2 were significantly (*p* < 0.05) higher than in mice immunized with S RBD alone, suggesting the improved ability of HBD 2-conjugated Ag to induce humoral immune responses (Figure 2A). Then we evaluated the ability of S RBD-HBD 2 to induce protective immunity against MERS-CoV infection by challenging immunized hDPP4-Tg mice with 10^5^ PFU MERS-CoV intranasally and monitoring their survival, weight, and pathological changes (Figure 2B,C). One-fourth of the mice intramuscularly immunized with S RBD-HBD 2 survived after intranasal MERS-CoV challenge infection. Although the mice immunized with S RBD-HBD 2 showed a moderate weight loss, their weight recovered rapidly after seven dpi. By contrast, control mice and mice immunized with S RBD alone exhibited a continuous weight loss, and they all died within 10 dpi.

**Figure 3:**
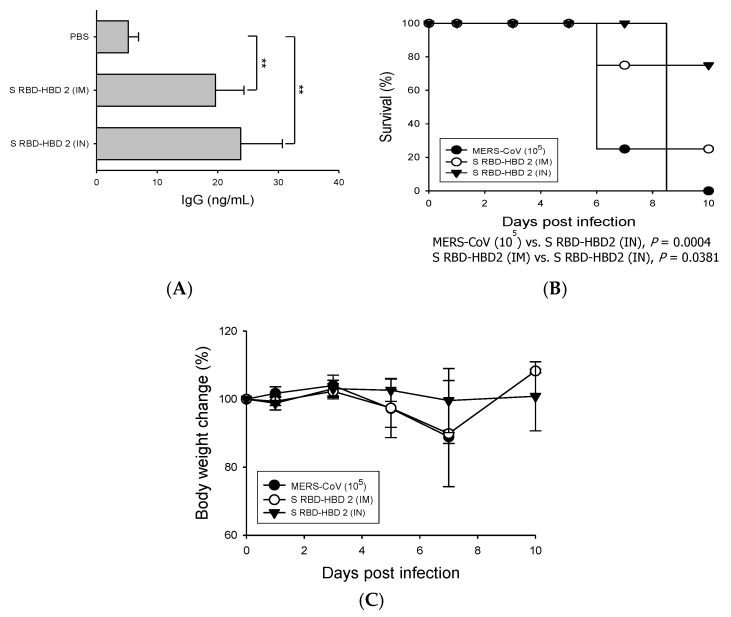
Intranasal immunization with S RBD-HBD 2 provides superior immunogenicity and protection against MERS-CoV in hDPP4-Tg mice. Mice were immunized with 5 µg/mouse of S RBD-HBD 2 intramuscularly (IM) or 1 µg/mouse intranasally (IN), and sera were collected three days after the last boost immunization. The levels of the S RBD-specific IgG were measured using ELISA. Data are presented as means ± SDs (*n* = 3). Immunized mice were challenged intranasally with MERS-CoV (10^5^ PFU) and monitored every other day for weight loss, clinical symptoms, and survival. (**A**) Serum levels of S RBD-specific IgG which were determined via capture ELISA using standard mouse IgG. ** *p* < 0.01. (**B**) Survival of hDPP4-Tg mice after MERS-CoV infection (*n* = 8). *P*-values were calculated using the log-rank (Mantel–Cox) test. (**C**) Body weight changes in MERS-CoV-infected mice. Results are presented as means ± SDs (*n* = 8) at the indicated times post-infection.

We also compared the ability of intranasal and intramuscular S RBD-HBD 2 immunization to induce S RBD-specific adaptive immune responses and protective immunity against MERS-CoV infection (Figure 3). For the comparative analysis, the results collected from the IM S RBD-HBD 2 immunization presented in Figure 2 were used for comparison. We found that the serum levels of S RBD-specific IgG were higher in intranasally immunized mice than in intramuscularly immunized mice (Figure 3A). Importantly, the survival rate after MERS-CoV infection was higher in hDPP4-Tg mice intranasally immunized with S RBD-HBD 2 than in intramuscularly immunized mice (75% vs. 25%; Figure 3B). In addition, we monitored the body weight changes in each group of mice following the virus challenge (Figure 3C). These results suggest that HBD 2 conjugation potentiates Ag-specific adaptive immune responses and protective immunity against viral infection.

### 3.3. HBD 2-Conjugated Ag Generates Strong Mucosal Iga Response and Prevents Lung Damage After MERS-CoV Infection

**Figure 4:**
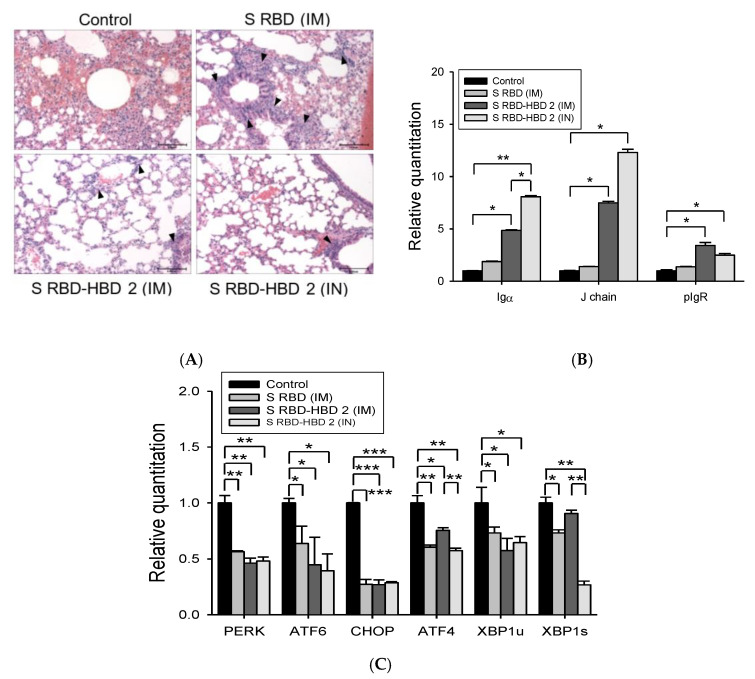
Immunization with S RBD-HBD 2 reinforces local mucosal Ab responses, downregulates ER-stress-associated genes, and prevents lung damage in hDPP4-Tg mice following MERS-CoV infection. (**A**) Representative H&E-stained lung sections from control and immunized hDPP4-Tg mice after MERS-CoV infection. H&E-stained lung sections were analyzed for inflammation by light microscopy. Control group represented distorted lung morphologies, including wider and thicker alveolar septa with perivascular and peribronchial cuffing. Immunized groups exhibited less alveolar thickening and fewer inflammatory infiltrations. Regions of inflammatory cell infiltration around vasculature, bronchiole, and proximal alveoli were noted by arrowheads. Scale bars = 100 µm. (**B**) Expression levels of SIgA-associated Igα chain, J chain, and pIgR in the lungs of hDPP4-Tg mice. Total RNA was extracted from the lung of hDPP4-Tg mice five days after infection with MERS-CoV and was used to assess the expression of mucosal IgA response-related genes by qRT-PCR. Reactions were performed in duplicate. Data are presented as means ± SD (*n* = 2). * *p* < 0.05 and ** *p* < 0.01. (**C**) Effect of S RBD immunization with or without HBD 2 on the expression of ER-stress-associated genes in the lungs of MERS-CoV infected hDPP4-Tg mice. Total RNA was extracted from the lungs of control and immunized mice five days after MERS-CoV infection and was used to assess the relative expression levels of ER-stress-associated genes by qRT-PCR. Reactions were performed in duplicate, and β-actin was used as the internal control for normalization. Fold changes relative to the non-treated controls are shown. Data are presented as means ± SD (*n* = 2). * *p <* 0.05, ** *p <* 0.01, and *** *p <* 0.001.

Next, we assessed the pathological changes in the lungs of MERS-CoV-challenged mice after different immunization regimens (Figure 4A). After the MERS-CoV challenge, the lungs of control mice exhibited marked pathological changes, including alveolar septal thickening, inflammatory cell infiltration, and hemorrhage. By contrast, the lungs of mice intramuscularly immunized with S RBD-HBD 2 had similar histopathological characteristics to those of normal mice after MERS-CoV infection, except modest changes in pneumocyte arrangement and alveolar septal thickening. Importantly, almost no histopathological changes were observed in the lungs of mice intranasally immunized with S RBD-HBD 2.

In the mucosa, the production of secretory IgA (SIgA) and polymeric Ig (pIg) receptor (pIgR)-mediated transport of SIgA plays an essential role in protecting against pathogen invasion and maintaining homeostasis in mucosal surfaces [23,24,25]. Furthermore, mucosal IgA production in vaccinated animals leads to memory response and long-term protection against various mucosal pathogens, including viruses, bacteria, and other intracellular pathogens [26]. Here, we explored the relevance of mucosal IgA responses in the protection against MERS-CoV infection. To this end, we analyzed the expression levels of Igα chain, J chain, and pIgR in the lungs of MERS-CoV-infected hDPP4-Tg mice after intranasal or intramuscular immunization with S RBD-HBD 2 because the SIgA levels in the lung depend on the production of IgA in the lamina propria and transport of pIgA-pIgR complex across epithelial cells (Figure 4B). hDPP4-Tg mice intranasally immunized with S RBD-HBD 2 exhibited high IgA levels in the lungs. Notably, the mRNA levels of Igα and J chains were significantly (*p* < 0.05) higher in hDPP4-Tg mice intranasally immunized with S RBD-HBD 2 than in intramuscularly immunized mice. Although pIgR mRNA levels were higher in the lungs of hDPP4-Tg mice intranasally immunized with S RBD-HBD 2 than in mice intramuscularly immunized with S RBD alone, they were slightly lower than in mice intramuscularly immunized with S RBD-HBD 2.

Interestingly, the ER-stress-associated genes *Perk*, *Atf4*, *Chop*, *Atf6*, and *Xbp1* were significantly less upregulated in the lungs of MERS-CoV-infected hDPP4-Tg mice after immunization with S RBD protein with or without HBD 2 (Figure 4C). Although there were no significant differences in the levels of these genes in the lungs of the mice immunized with S RBD-HBD 2 intramuscularly or intranasally, *Aft4* and *Xbp1s* were expressed in significantly (*p* < 0.01) lower levels in the lungs of intranasally immunized mice. These results suggest that HBD2-conjugation induces potent mucosal IgA responses, preventing ER-stress-mediated lung damage following MERS-CoV infection.

### 3.4. Intranasal S RBD-HBD 2 Administration Induces Potent Antiviral Immune Responses Protecting hDPP4-Tg Mice against MERS-CoV Infection

**Figure 5:**
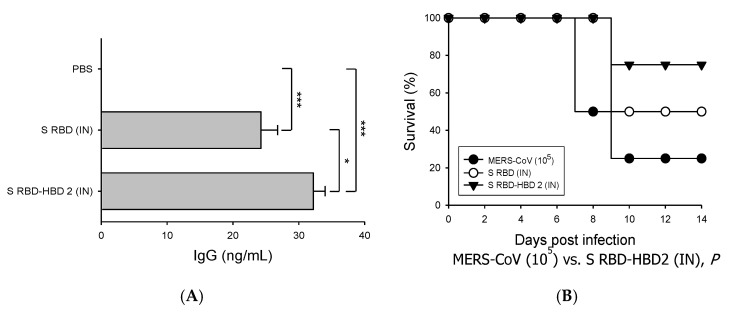

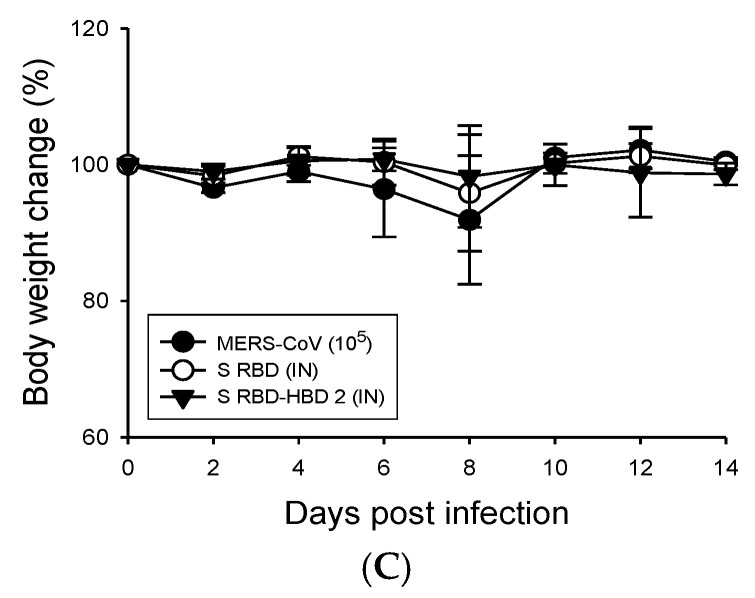
Intranasal administration of S RBD-HBD 2 induces potent systemic antibody responses, preventing MERS-CoV infection in hDPP4-Tg mice. Mice were intranasally (IN) administered with 1 µg/mouse of S RBD with or without HBD 2, and sera were collected three days after the last boost immunization. The serum levels of the S-RBD-specific IgG were measured using ELISA. Data are presented as means ± SDs (*n* = 3). Immunized mice were challenged intranasally with MERS-CoV (10^5^ PFU). Infected mice were monitored every other day for weight loss, clinical symptoms, and survival. (**A**) Serum levels of S-RBD-specific IgG which were determined via capture ELISA using standard mouse IgG. * *p* < 0.05 and *** *p* < 0.001. (**B**) Survival of hDPP4-Tg mice after MERS-CoV infection (*n* = 8). *p*-values were calculated using the log-rank (Mantel–Cox) test. (**C**) Body weight changes in MERS-CoV-infected mice. Results are presented as means ± SDs (*n* = 8) at the indicated times post-infection.

We also evaluated the ability of low-dose S RBD-HBD 2 intranasal administration to induce Ag-specific Ab responses and protect hDPP4-Tg mice against intranasal challenge with MERS-CoV (Figure 5). The serum levels of S RBD-specific IgG were significantly higher in mice intranasally immunized with S RBD-HBD 2 than in control mice or mice immunized with S RBD alone (*p* < 0.001 and *p* < 0.05; Figure 5A). Importantly, S-RBD-HBD-2-immunized mice exhibited a markedly improved survival rate (75%) at 10 dpi with MERS-CoV compared to control mice (25%) and mice immunized with S RBD alone (Figure 5B). In contrast to S RBD alone- and S-RBD-HBD-2-immunized mice, MERS-CoV-infected control mice showed a modest weight loss until eight dpi (Figure 5C). No profound weight loss was observed in S RBD alone- and S-RBD-HBD-2-immunized hDPP4-Tg mice following MERS-CoV infection. These results suggest that the intranasal immunization of S RBD-HBD 2 induces potent mucosal IgA responses, reinforcing local and systemic immunity, and preventing MERS-CoV infection.

## 4. Discussion

MERS-CoV has spread in 27 countries since the 2012 outbreak in Saudi Arabia; it infects the lower respiratory tract of humans, causing acute respiratory distress syndrome with approximately 34.5% mortality [27]. Currently, there are no therapies or vaccines against MERS-CoV on the market, and only a few candidates are being tested in a clinical setting. Although inactivated viruses and vector-based vaccines induce strong cellular and humoral immune responses, their safety remains a matter of debate [28,29]. Preexisting vector-specific immunity due to natural infections can lead to devastating host immune response, limiting the use of vector-based vaccines [30,31]. Vaccination strategies with S protein or S RBD subunits have been proven to be more effective and safer than vector-based vaccine candidates against SARS-CoV [32,33]. However, S protein is metastable and difficult to produce recombinantly. In order to achieve a high-yield production of recombinant S protein, there was a study to increase the stability of the protein through proline substitution of S protein [34]. Notably, intramuscular administration of an RBD-based vaccine in mice induces long-term protection against the SARS-CoV infection [35]. Three doses of intramuscularly administered recombinant S-NTD also induces protective immunity against MERS-CoV infection in Ad5-hDPP4 mice [36]. Besides the intramuscular route, vaccines can be administered via the subcutaneous, intradermal, intragastric, and intranasal routes [37]. Importantly, the intranasal administration of an RBD-based subunit vaccine induces strong mucosal IgA responses [18]. In addition, prime-boost immunization using a full-length S DNA vaccine and an S1 protein boost induces virus-neutralizing antibodies and confers protection against MERS-CoV infection in non-human primate models [38]. Given that different types of MERS-CoV vaccine candidates have variable degrees of immunogenicity, the combination of the optimal adjuvant and immunization strategy, rather than immunogen-only regimens, could enhance immunogenicity and protective immune responses against viral infection.

In this study, we investigated the potential use of HBD 2 as an adjuvant to strengthen Ag-specific adaptive immune responses in hDPP4-Tg mice (Figure 2). HBD 2 is a cysteine-rich cationic low-molecular-weight antimicrobial peptide. In addition to their function as host defense peptides against various pathogens, β-defensins share structural similarity with chemokines and play a critical role as chemoattractants, recruiting immune cells to mucosal sites and thereby linking innate and adaptive immunity [12,39]. In response to pathogenic invasion, DCs found in mucosal tissues orchestrate immune responses against the invading pathogens [40]. Moreover, B cells expressing defensins contribute to a prolonged cellular and humoral immune response to infected pathogens [41]. Consequently, β-defensin has been considered a useful tool for improving systemic and mucosal immune responses of subunit vaccines by recruiting antigen-presenting cells and priming adaptive immune responses [42]. We previously found that HBD 2 promotes antiviral innate immune responses in macrophage-like THP-1 cells and enhances the production of virus-specific neutralizing Abs in vivo [15,16].

The role of mucosal immunity in the lungs and other primary infectious tissues has not been widely studied, although the respiratory mucosa is the main infectious site for numerous pathogens, including MERS-CoV. In this study, we intramuscularly or intranasally immunized hDPP4-Tg mice with S RBD protein with or without HBD 2 and investigated systemic and mucosal immune responses against MERS-CoV. Ag-specific Ab levels and protective immunity against intranasal viral infection were significantly potentiated in mice intranasally immunized with S RBD-HBD 2, although the Ag dose was lower than the dose used for intramuscular immunization (Figure 3). It is worth noting that the levels of Ag-specific Abs and protective immunity against MERS-CoV did not significantly differ in hDPP4-Tg mice intranasally immunized with a low (1 µg/mouse) or high (5 µg/mouse) S RBD-HBD 2 dose (data not shown). Intramuscular immunization with a low dose of S RBD protein with or without HBD 2 elicited weak protective and Ag-specific IgG responses in compare to intranasal immunization (data not shown). By contrast, intranasal administration of S RBD-HBD 2 elicited potent systemic and mucosal Ab responses, protecting against MERS-CoV infection (Figure 5). These data suggest that the use of HBD 2 as an adjuvant enhances local mucosal and systemic immune responses, particularly when administered intranasally. We also demonstrated that MERS-CoV infection triggered ER stress pathways promoting pulmonary fibrosis (Figure 1). MERS-CoV infection in the respiratory tract causes fibrotic lung disease symptoms, including alveolar cell damage, inflammation, fibroblast proliferation, and extracellular matrix deposition [43]. However, the mechanisms underlying progressive lung fibrosis following MERS-CoV infection remain elusive.

ER stress has been implicated in fibrotic remodeling by activating unfolded protein responses and pro-apoptotic pathways, as well as promoting inflammation in various tissues, including the lungs, liver, gastrointestinal tract, kidney, and heart. We previously demonstrated that, upon MERS-CoV infection, tissue damage elicits severe inflammatory responses and activate ER stress pathway components, including PERK, ATF4, CHOP, and ATF6. In this study, we showed that the administration of HBD 2-conjugated S RBD downregulated various ER stress-associated molecules and alleviated progressive pulmonary fibrosis in the lungs of MERS-CoV-infected hDPP4-Tg mice (Figure 4).

## 5. Conclusions

Overall, these results suggest that the conjugation of HBD 2 with S RBD protein enhances systemic and mucosal immune responses that protect from MERS-CoV infection. Moreover, these findings indicate that immunization via the intranasal route might be superior in triggering protective local and systemic immunity against mucosal pathogens. Therefore, the use of HBD 2 as an adjuvant may represent a promising approach to enhance the immunogenicity and safety of subunit vaccine candidates against MERS-CoV and other mucosal viruses.

## Figures and Tables

**Table 1:** Sequences of the qRT-PCR primers. Primers used to measure the expression levels of genes associated with endoplasmic reticulum (ER)-stress and IgA immune responses. *β-actin* was used as an endogenous control.

Gene	Primer Sequence
PERK	F: 5′-AGT CCC TGC TCG AAT CTT CCT-3′
	R: 5′-TCC CAA GGC AGA ACA GAT ATA CC-3′
ATF4	F: 5′-TCC TGA ACA GCG AAG TGT TG -3′
	R: 5′-ACC CAT GAG GTT TCA AGT GC -3′
ATF6	F: 5′-TGC CTT GGG AGT CAG ACC TAT-3′
	R: 5′-GCT GAG TTG AAG AAC ACG AGT C-3′
CHOP	F: 5′-CTG GAA GCC TGG TAT GAG GAT-3′
	R: 5′-CAG GGT CAA GAG TAG TGA AGG T-3′
XBP1u	F: 5′-AAG AAC ACG CTT GGG AAT GG-3′
	R: 5′-ACT CCC CTT GGC CTC CAC-3′
XBP1s	F: 5′-GAG TCC GCA GCA GGT G-3′
	R: 5′-GTG TCA GAG TCC ATG GGA-3′
Igα chain	F: 5′-CGT CCA AGA ATT GGA TGT GA-3′
	R: 5′-AGT GAC AGG CTG GGA TGG-3′
J chain	F: 5′-GAA CTT TGT ATA CCA TTT GTC AGA CG-3′
	R: 5′-CTG GGT GGC AGT AAC AAC CT-3′
pIgR	F: 5′-AGT AAC CGA GGC CTG TCC TT-3′
	R: 5′-GTC ACT CGG CAA CTC AGG A-3′
β-actin	F: 5′-CGT ACC ACA GGC ATT GTG A-3′
	R: 5′-CTC GTT GCC AAT AGT GAT GA-3′

F and R: sequences of the forward and reverse primers, respectively.

## References

[1] Kim K.H., Tandi T.E., Choi J.W., Moon J.M., Kim M.S. (2017). Middle East respiratory syndrome (MERS-CoV) outbreak in South Korea: Epidemiology, characteristics and public health implications. J. Hosp. Infect..

[2] World Health Organization (2019). Countries Agree Next Steps to Combat Global Health Threat by MERS-CoV.

[3] Schindewolf C., Menachery V.D. (2019). Middle East respiratory syndrome vaccine candidates: Cautious optimism. Viruses.

[4] Modjarrad K., Moorthy V.S., Ben Embarek P., Van Kerkhove M., Kim J., Kieny M.P. (2016). A roadmap for MERS-CoV research and product development: Report from a World Health Organization consultation. Nat. Med..

[5] Okba N.M., Raj V.S., Haagmans B.L. (2017). Middle East respiratory syndrome coronavirus vaccines: Current status and novel approaches. Curr. Opin. Virol..

[6] Zhang N., Channappanavar R., Ma C., Wang L., Tang J., Garron T., Tao X., Tasneem S., Lu L., Tseng C.T. (2016). Identification of an ideal adjuvant for receptor-binding domain-based subunit vaccines against Middle East respiratory syndrome coronavirus. Cell. Mol. Immunol..

[7] Coleman C.M., Liu Y.V., Mu H., Taylor J.K., Massare M., Flyer D.C., Glenn G.M., Smith G.E., Frieman M.B. (2014). Purified coronavirus spike protein nanoparticles induce coronavirus neutralizing antibodies in mice. Vaccine.

[8] Podda A., Del Giudice G. (2003). MF59-adjuvanted vaccines: Increased immunogenicity with an optimal safety profile. Expert Rev. Vaccines.

[9] Jiang S., Bottazzi M.E., Du L., Lustigman S., Tseng C.T., Curti E., Jones K., Zhan B., Hotez P.J. (2012). Roadmap to developing a recombinant coronavirus S protein receptor-binding domain vaccine for severe acute respiratory syndrome. Expert Rev. Vaccines.

[10] Boniotto M., Jordan W.J., Eskdale J., Tossi A., Antcheva N., Crovella S., Connell N.D., Gallagher G. (2006). Human beta-defensin 2 induces a vigorous cytokine response in peripheral blood mononuclear cells. Antimicrob. Agents Chemother..

[11] Pazgier M., Hoover D.M., Yang D., Lu W., Lubkowski J. (2006). Human beta-defensins. Cell. Mol. Life Sci..

[12] Yang D., Chertov O., Bykovskaia S.N., Chen Q., Buffo M.J., Shogan J., Anderson M., Schröder J.M., Wang J.M., Howard O.M. (1999). Beta-defensins: Linking innate and adaptive immunity through dendritic and T cell CCR6. Science.

[13] Biragyn A., Belyakov I.M., Chow Y.H., Dimitrov D.S., Berzofsky J.A., Kwak L.W. (2002). DNA vaccines encoding human immunodeficiency virus-1 glycoprotein 120 fusions with proinflammatory chemoattractants induce systemic and mucosal immune responses. Blood.

[14] Biragyn A., Ruffini P.A., Leifer C.A., Klyushnenkova E., Shakhov A., Chertov O., Shirakawa A.K., Farber J.M., Segal D.M., Oppenheim J.J. (2002). Toll-like receptor 4-dependent activation of dendritic cells by beta-defensin 2. Science.

[15] Kim J., Yang Y.L., Jang S.H., Jang Y.S. (2018). Human β-defensin 2 plays a regulatory role in innate antiviral immunity and is capable of potentiating the induction of antigen-specific immunity. Virol. J..

[16] Kim J., Yang Y.L., Jang Y.S. (2019). Human β-defensin 2 is involved in CCR2-mediated Nod2 signal transduction, leading to activation of the innate immune response in macrophages. Immunobiology.

[17] Kim J., Yang Y.L., Jeong Y.S., Jang Y.S. (2020). Middle East respiratory syndrome-coronavirus infection into established hDPP4-transgenic mice accelerates lung damage via activation of the pro-inflammatory response and pulmonary fibrosis. J. Microbiol. Biotechnol..

[18] Ma C., Wang L., Tao X., Zhang N., Yang Y., Tseng C.K., Li F., Zhou Y., Jiang S., Du L. (2014). Searching for an ideal vaccine candidate among different MERS coronavirus receptor-binding fragments: The importance of immunofocusing in subunit vaccine design. Vaccine.

[19] Cockrell A.S., Peck K.M., Yount B.L., Agnihothram S.S., Scobey T., Curnes N.R., Baric R.S., Heise M.T. (2014). Mouse dipeptidyl peptidase 4 is not a functional receptor for Middle East respiratory syndrome coronavirus infection. J. Virol..

[20] Pahl H.L. (1999). Signal transduction from the endoplasmic reticulum to the cell nucleus. Physiol. Rev..

[21] Burman A., Tanjore H., Blackwell T.S. (2018). Endoplasmic reticulum stress in pulmonary fibrosis. Matrix Biol..

[22] Wang C., Zheng X., Gai W., Zhao Y., Wang H., Wang H., Feng N., Chi H., Qiu B., Li N. (2017). MERS-CoV virus-like particles produced in insect cells induce specific humoural and cellular immunity in rhesus macaques. Oncotarget.

[23] Johansen F.E., Braathen R., Brandtzaeg P. (2001). The J chain is essential for polymeric Ig receptor-mediated epithelial transport of IgA. J. Immunol..

[24] Shimada S., Kawaguchi-Miyashita M., Kushiro A., Sato T., Nanno M., Sako T., Matsuoka Y., Sudo K., Tagawa Y., Iwakura Y. (1999). Generation of polymeric immunoglobulin receptor-deficient mouse with marked reduction of secretory IgA. J. Immunol..

[25] Uren T.K., Johansen F.E., Wijburg O.L., Koentgen F., Brandtzaeg P., Strugnell R.A. (2003). Role of the polymeric Ig receptor in mucosal B cell homeostasis. J. Immunol..

[26] Liew F.Y., Russell S.M., Appleyard G., Brand C.M., Beale J. (1984). Cross-protection in mice infected with influenza A virus by the respiratory route is correlated with local IgA antibody rather than serum antibody or cytotoxic T cell reactivity. Eur. J. Immunol..

[27] Xu J., Jia W., Wang P., Zhang S., Shi X., Wang X., Zhang L. (2019). Antibodies and vaccines against Middle East respiratory syndrome coronavirus. Emerg. Microbes Infect..

[28] Deng Y., Lan J., Bao L., Huang B., Ye F., Chen Y., Yao Y., Wang W., Qin C., Tan W. (2018). Enhanced protection in mice induced by immunization with inactivated whole viruses compare to spike protein of Middle East respiratory syndrome coronavirus. Emerg. Microbes Infect..

[29] Wang L., Cheng W., Zhang Z. (2017). Respiratory syncytial virus infection accelerates lung fibrosis through the unfolded protein response in a bleomycin-induced pulmonary fibrosis animal model. Mol. Med. Rep..

[30] Song F., Fux R., Provacia L.B., Volz A., Eickmann M., Becker S., Osterhaus A.D., Haagmans B.L., Sutter G. (2013). Middle East respiratory syndrome coronavirus spike protein delivered by modified vaccinia virus Ankara efficiently induces virus-neutralizing antibodies. J. Virol..

[31] Pichla-Gollon S.L., Lin S.W., Hensley S.E., Lasaro M.O., Herkenhoff-Haut L., Drinker M., Tatsis N., Gao G.P., Wilson J.M., Ertl H.C. (2009). Effect of preexisting immunity on an adenovirus vaccine vector: In vitro neutralization assays fail to predict inhibition by antiviral antibody in vivo. J. Virol..

[32] Du L., He Y., Zhou Y., Liu S., Zheng B.J., Jiang S. (2009). The spike protein of SARS-CoV: A target for vaccine and therapeutic development. Nat. Rev. Microbiol..

[33] He Y., Lu H., Siddiqui P., Zhou Y., Jiang S. (2005). Receptor-binding domain of severe acute respiratory syndrome coronavirus spike protein contains multiple conformation-dependent epitopes that induce highly potent neutralizing antibodies. J. Immunol..

[34] Hsieh C.L., Goldsmith J.A., Schaub J.M., DiVenere A.M., Kuo H.C., Javanmardi K., Le K.C., Wrapp D., Lee A.G., Liu Y. (2020). Structure-based design of prefusion-stabilized SARS-CoV-2 spikes. Science.

[35] Du L., Zhao G., He Y., Guo Y., Zheng B.J., Jiang S., Zhou Y. (2007). Receptor-binding domain of SARS-CoV spike protein induces long-term protective immunity in an animal model. Vaccine.

[36] Jiaming L., Yanfeng Y., Yao D., Yawei H., Linlin B., Baoying H., Jinghua Y., Gao G.F., Chuan Q., Wenjie T. (2017). The recombinant N-terminal domain of spike proteins is a potential vaccine against Middle East respiratory syndrome coronavirus (MERS-CoV) infection. Vaccine.

[37] Guo X., Deng Y., Chen H., Lan J., Wang W., Zou X., Hung T., Lu Z., Tan W. (2015). Systemic and mucosal immunity in mice elicited by a single immunization with human adenovirus type 5 or 41 vector-based vaccines carrying the spike protein of Middle East respiratory syndrome coronavirus. Immunology.

[38] Wang L., Shi W., Joyce M.G., Modjarrad K., Zhang Y., Leung K., Lees C.R., Zhou T., Yassine H.M., Kanekiyo M. (2015). Evaluation of candidate vaccine approaches for MERS-CoV. Nat. Commun..

[39] Röhrl J., Yang D., Oppenheim J.J., Hehlgans T. (2010). Human beta-defensin 2 and 3 and their mouse orthologs induce chemotaxis through interaction with CCRJ2. J. Immunol..

[40] Coombes J.L., Powrie F. (2008). Dendritic cells in intestinal immune regulation. Nat. Rev. Immunol..

[41] Allaker R.P. (2008). Host defence peptides: A bridge between the innate and adaptive immune responses. Trans. R. Soc. Trop. Med. Hyg..

[42] Hand T.W. (2016). The role of the microbiota in shaping infectious immunity. Trends Immunol..

[43] Moore B.B., Hogaboam C.M. (2008). Murine models of pulmonary fibrosis. Am. J. Physiol..

